# Distribution-based covariate assessment using wasserstein distance in population pharmacokinetic models

**DOI:** 10.3389/fphar.2026.1804989

**Published:** 2026-07-06

**Authors:** Nicolas Simon, Jean-Sebastien Hulot, Katharina von Fabeck

**Affiliations:** 1 Department of Clinical Pharmacology, APHM, Institut de Neurosciences de la Timone, UMR7289, CNRS, Hôpital Sainte Marguerite, CAP-TV, Aix Marseille University, Marseille, France; 2 Université Paris Cité, INSERM, PARCC, Paris, France; 3 CIC1418, AP-HP, Hôpital Européen Georges-Pompidou, Paris, France

**Keywords:** covariate model validation, methotrexate, model-informed precision dosing, optimal transport, population pharmacokinetics, Wasserstein distance

## Abstract

**Background:**

Population pharmacokinetic modeling relies on adequate covariate specification to explain interindividual variability and support model-informed precision dosing. Current validation methods predominantly use likelihood-based criteria and correlation analyses, which may fail to detect complex non-linear or non-monotonic covariate relationships. We propose the Wasserstein distance, derived from optimal transport theory, as a complementary distribution-based metric for covariate specification assessment.

**Methods:**

Using high-dose methotrexate pharmacokinetic data from 50 patients with lymphoid malignancies, we developed three nested population models: BASE (no covariates), GENET_ALL (ABCC2 -24C>T polymorphism), and FINAL (genetic plus creatinine clearance). We computed Wasserstein distances between empirical distributions of individual parameter deviations stratified by covariate groups, with statistical significance assessed via permutation testing. We defined a Wasserstein-based R^2^ metric to quantify variance reduction across models. Simulation studies illustrated detection of threshold, U-shaped, and Simpson’s paradox-like covariate effects.

**Results:**

In the FINAL model, both CT01 (W = 0.071, p = 0.741) and creatinine clearance showed no residual distributional differences, confirming adequate specification. The Wasserstein-based R^2^ demonstrated progressive improvement (GENET_ALL: 21.5%, FINAL: 31.7% reduction). Simulations illustrated the complementary value of the Wasserstein-based approach alongside classical methods, particularly for detecting complex non-linear relationships.

**Conclusion:**

Wasserstein distance provides a complementary distribution-based framework with limited parametric assumptions for validating covariate specification in population pharmacokinetic models, provided that eta-shrinkage remains sufficiently low when using empirical Bayes estimates. This approach is particularly useful for detecting complex non-linear relationships.

## Highlights

- This study introduces a new way to check whether patient characteristics are correctly accounted for in population pharmacokinetic models, by comparing how individual responses are distributed across patient groups.
- Applied to high-dose methotrexate, this approach confirmed that genetic and renal factors were appropriately handled in the final model and helped quantify how much unexplained variability was reduced.
- By using realistic simulations, the method proved particularly useful for revealing complex or non-obvious relationships that standard statistical approaches may overlook, supporting more reliable model-informed dosing decisions.

## 1 Introduction

Population pharmacokinetic (PopPK) modeling has become an essential tool in drug development and therapeutic drug monitoring, allowing researchers to characterize drug disposition in target populations and identify sources of interindividual variability (Mould and Upton, 2012; Bauer, 2019). A critical component of PopPK model development is covariate analysis, which aims to explain variability in pharmacokinetic parameters through patient-specific characteristics such as renal function, body weight, age, or genetic polymorphisms (Ribbing and Jonsson, 2004). The identification and adequate specification of relevant covariates is fundamental not only for understanding the determinants of drug exposure but also for enabling model-informed precision dosing in clinical practice (Darwich et al., 2021).

Traditional approaches to covariate model evaluation rely primarily on likelihood-based statistical criteria such as the change in objective function value, combined with visual inspection of goodness-of-fit plots and residual analyses (Byon et al., 2013). These methods typically assess whether the inclusion of a covariate significantly improves model fit and reduces unexplained interindividual variability. However, statistical significance does not always translate to adequate covariate specification. A covariate may reach statistical significance while still failing to fully capture the relationship between the patient characteristic and the pharmacokinetic parameter, leaving remaining correlation that could affect model predictions and precision dosing recommendations.

The assessment of covariate adequacy can be conceptualized as a distributional comparison problem. If a covariate is adequately specified in the model, the remaining variability, represented by individual deviations from population predictions (eta values), should show no systematic relationship with that covariate (Savic and Karlsson, 2009). In other words, the distributions of individual parameter estimates (Empirical Bayes Estimates) should not differ significantly across covariate groups after accounting for the covariate effect in the structural model. Classical approaches to test this hypothesis rely on correlation coefficients between individual parameters and covariates. However, correlation-based methods present important limitations, particularly in scenarios involving threshold effects, non-monotonic relationships, or Simpson’s paradox, where Pearson correlation may approach zero or even reverse sign despite clear functional relationships.

The Wasserstein distance, also known as the Earth Mover’s Distance, offers a promising alternative for assessing distributional differences in the context of covariate validation (Villani, 2008; Peyré and Cuturi, 2019). Originating from optimal transport theory, this metric quantifies the minimal cost of transforming one probability distribution into another. Unlike divergence measures such as the Kullback-Leibler divergence, the Wasserstein distance provides a global assessment that accounts for location, dispersion, and shape of distributions without requiring normality assumptions (Kolouri et al., 2017). This metric has gained increasing adoption across diverse fields including machine learning, genomics, and biostatistics (Schiebinger et al., 2019).

For univariate distributions, the first-order Wasserstein distance has a closed-form solution based on the integral of the absolute difference between cumulative distribution functions, making it computationally efficient and readily interpretable (Givens and Shortt, 1984). In the PopPK context, this property can be leveraged to compare distributions of individual pharmacokinetic parameters or their deviations between different covariate groups. A small Wasserstein distance would indicate similar distributions, supporting adequate covariate specification, while a large distance would suggest systematic differences that warrant further model refinement. The application of Wasserstein distance to covariate specification testing in population pharmacokinetics, however, remains largely unexplored. We hypothesize that this metric, combined with permutation-based statistical testing, provides a complementary and flexible method for validating whether covariates are adequately captured in PopPK models, particularly in scenarios where traditional correlation-based approaches may fail.

In this study, we demonstrate the application of Wasserstein distance for covariate validation using a clinical dataset of high-dose methotrexate pharmacokinetics in pediatric patients with lymphoid malignancies. Methotrexate exhibits substantial interindividual variability in clearance and volume of distribution, partially explained by renal function and genetic polymorphisms in drug transporters, particularly ABCC2 (Simon et al., 2013). Using data from Simon et al. (Simon et al., 2013), we developed three nested PopPK models incorporating progressively more covariates: a base model without covariates, a genetic model including the ABCC2 -24C>T polymorphism, and a final model incorporating both genetic and physiological covariates (creatinine clearance). Following the methodological framework proposed by Taylor et al. (Taylor et al., 2023) for model selection and validation, we applied permutation-based Wasserstein distance testing to assess whether each covariate was adequately specified by comparing distributions of individual parameter deviations across covariate groups.

We further illustrate the advantages of this approach through simulation studies designed to highlight scenarios where Wasserstein distance may provide complementary information to classical correlation-based methods, including threshold effects and U-shaped covariate relationships. The objectives of this work are threefold: first, to present the theoretical foundation and statistical framework for applying Wasserstein distance to covariate validation; second, to demonstrate its practical implementation through methotrexate pharmacokinetic data analysis; and third, to establish a reproducible methodological workflow that can be applied to other pharmacometric analyses. This work contributes to improving model validation procedures in population pharmacokinetics, ultimately supporting more reliable model-informed precision dosing in clinical practice.

## 2 Materials and methods

### 2.1 Clinical dataset

We analyzed pharmacokinetic data from patients with lymphoid malignancies treated with high-dose methotrexate. Individual pharmacokinetic parameters were derived from a previously published population pharmacokinetic model developed by Simon et al. (Simon et al., 2013). The dataset consisted of 50 patients for whom individual pharmacokinetic parameters were available from a previously developed population pharmacokinetic model. Individual parameters were derived as empirical Bayes estimates (ETAs) obtained using NONMEM software and included clearance (CL) and central volume of distribution (V1), together with their associated between-subject variability. ETAs were used in this study as conditional summaries of individual deviations from population-level pharmacokinetic parameters. Although empirical Bayes estimates are subject to shrinkage and are not independent observations, their empirical distributions may provide a practical representation of residual interindividual variability after accounting for modeled covariate effects, provided that eta-shrinkage is sufficiently low. Details of the study design, population, and pharmacokinetic model development are described in the original publication (Simon et al., 2013). The study was approved by the Ethics Committee of Pitie-Salpetriere Hospital, Paris, France, according to the requirement of the French law on clinical research.

### 2.2 Population pharmacokinetic models

Three nested population pharmacokinetic models were evaluated to assess covariate specification adequacy:

- BASE model: No covariates included. This model describes population-level pharmacokinetic parameters with unexplained between-subject variability only.
- GENET_ALL model: Inclusion of a genetic covariate on MTX clearance. The genetic covariate corresponds to the ABCC2 −24C>T polymorphism (rs717620), hereafter referred to as CT01 for simplicity. This model accounts for the effect of genetic variability on MTX elimination.
- FINAL model: Inclusion of both the genetic covariate (CT01) and creatinine clearance (CLCR) on MTX clearance. This model represents the most complete covariate structure, accounting for both genetic and physiological determinants of MTX pharmacokinetics. Genetic effects were modeled multiplicatively, while creatinine clearance was included as an additive covariate on clearance.

The population pharmacokinetic model was a three-compartment model with first-order elimination developed in the original publication (Simon et al., 2013). The original population pharmacokinetic model included both interindividual variability and residual unexplained variability. The covariate relationships for clearance in the three nested models were specified as follows:

BASE model:

$$\text{CL}\_i=\text{CL}\_\text{pop }x\;\exp\left(\eta \_\text{CL},i\right)$$

GENET_ALL model:

$$\text{CL}\_i=\text{CL}\_\text{pop }x\;\left(\theta \_\text{CT}01\;**\text{ CT}01\_i\right)\;x\;\exp\left(\eta \_\text{CL},i\right)$$

$$V1\_i=V1\_\text{pop }x\;\left(\theta \_\text{CT}01\;**\text{ CT}01\_i\right)\;x\;\exp\left(\eta \_V1,i\;\right)$$

FINAL model:

CL_i=CL_pop x CLCR_i/89^θ_CLCR xθ_CT01 ** CT01_i x expη_CL,i

$$V1\_i=V1\_\text{pop }x\;\left(\theta \_\text{CT}01\;**\text{ CT}01\_i\right)\;x\;\exp\left(\eta \_V1,i\;\right)$$

where CL_pop represents the population typical clearance, CT01_i is a binary indicator (0 or 1) for the ABCC2 -24C>T polymorphism, CLCR_i is creatinine clearance standardized to 89 mL/min, V1_pop represents the population typical central volume of distribution, θ parameters represent fixed effects, and η_i represents individual random effects with variance ω^2^. Parameter estimates are reported in the original publication (Simon et al., 2013).

The objective of this step was to evaluate whether progression from the BASE model to the GENET_ALL and FINAL models resulted in improved covariate specification, as assessed by reductions in residual distributional differences.

### 2.3 Wasserstein distance calculation

The Wasserstein distance, also known as the Earth Mover’s Distance, quantifies the dissimilarity between two probability distributions by measuring the minimum cost required to transform one distribution into another (Villani, 2008; Peyré and Cuturi, 2019; Kolouri et al., 2017). For two univariate probability distributions (P) and (Q) with cumulative distribution functions (F_P) and (F_Q), the first-order Wasserstein distance is defined as:

$$W_{1}\left(P,Q\right)=\int \left|F\_P\left(x\right)\;‐\;F\_Q\left(x\right)\right|\mathrm{dx}$$

This metric captures differences in location, dispersion, and shape without relying on parametric assumptions or normality. In the present work, Wasserstein distances were computed between empirical cumulative distribution functions (ECDFs) of ETA values stratified by covariate groups.

### 2.4 Covariate analysis on real data

#### 2.4.1 Genetic covariate analysis (CT01 polymorphism)

The CT01 genetic polymorphism (ABCC2 −24C>T) was analyzed as a binary covariate (presence versus absence of at least one T allele) affecting MTX clearance. ETA distributions for clearance (ETA_CL) were compared between CT01-positive and CT01-negative patients using two approaches:

- Classical methods: Student’s t-test and Pearson correlation between ETA_CL and CT01 status.
- Wasserstein-based approach: Permutation testing using the Wasserstein distance. The observed Wasserstein distance between CT01 groups was compared to a null distribution generated by randomly permuting group labels (10,000 iterations).

Under adequate covariate specification, residual variability should be independent of the covariate, resulting in no significant distributional differences in ETA values between groups.

#### 2.4.2 Creatinine clearance analysis (CLCR)

Creatinine clearance was analyzed as a continuous covariate affecting MTX clearance. Patients were stratified into quartiles based on CLCR values. ETA_CL distributions were compared across quartiles using:

- Classical methods: Analysis of variance (ANOVA) and linear regression (ETA_CL as a function of CLCR).
- Wasserstein-based approach: Pairwise permutation tests between CLCR quartiles, with adjustment for multiple comparisons.

This strategy allowed assessment of residual distributional differences across physiological strata after covariate inclusion in the model. Quartiles were chosen as a pragmatic and commonly used approach for exploratory stratification of continuous covariates; however, alternative partitions (e.g., quintiles or data-driven groupings) could be considered and may influence the sensitivity of the analysis.

#### 2.4.3 Wasserstein-based R^2^ metric

To quantify the relative reduction in residual distributional differences associated with covariate inclusion, a Wasserstein-based coefficient was defined as:

$$R^{2}\_W=1\;‐\;\left(W\_\text{covariate}\_\text{model }/\;W\_\text{base}\_\text{model}\right)$$

where (W_base_model) denotes the Wasserstein distance computed from the BASE model, and (W_covariate_model) denotes the corresponding distance from models including covariates (GENET_ALL or FINAL). For each model, W_to_zero was defined as the first-order Wasserstein distance between the empirical distribution of ETA_CL and a degenerate reference distribution centered at zero, thereby quantifying the overall dispersion of individual deviations from the population prediction. Model comparison was performed at the level of individual random effects for clearance. Wasserstein-based metrics quantify how much unexplained variability remains for a given parameter, rather than assessing overall model fit. Smaller W_to_zero values indicate residuals more tightly centered around zero, reflecting improved model specification. This metric is not intended as a direct analogue of the classical coefficient of determination but rather as a normalized measure of reduction in distributional discrepancy attributable to covariate modeling.

#### 2.4.4 Simulation studies: non-linear covariate relationships

To illustrate scenarios in which Wasserstein distance may provide complementary information to classical correlation-based methods, simulation studies were conducted using a synthetic covariate representing serum albumin concentration. Albumin values were simulated from a normal distribution (mean 40 g/L, standard deviation 5 g/L) in a simulated population of 50 individuals matching the sample size of the clinical dataset.

#### 2.4.5 Scenario A: threshold effect (Hypoalbuminemia)

A threshold effect was simulated whereby patients with albumin concentrations below 35 g/L exhibited reduced MTX clearance:

- Albumin < 35 g/L: ETA_CL ∼ N (0.45, 0.12)
- Albumin ≥ 35 g/L: ETA_CL ∼ N (0.00, 0.12)

This scenario mimics clinically relevant step-function relationships, such as those arising from protein binding or physiological reserve.

#### 2.4.6 Scenario B: U-shaped relationship

A U-shaped relationship was simulated to represent complex non-linear effects:

- Albumin < 35 g/L: ETA_CL ∼ N (0.40, 0.12)
- Albumin 35–45 g/L: ETA_CL ∼ N (0.00, 0.12)
- Albumin > 45 g/L: ETA_CL ∼ N (0.35, 0.12)

Such patterns reflect physiological situations in which deviations from an optimal range in either direction influence pharmacokinetics.

#### 2.4.7 Scenario C: Simpson’s paradox (Latent confounding and aggregation bias)

A third simulation scenario was designed to illustrate a Simpson’s paradox–like situation, in which marginal association measures fail due to aggregation over an unobserved confounding structure. A continuous covariate analogous to serum albumin was simulated together with an unobserved binary grouping variable representing a latent patient characteristic influencing both the covariate distribution and pharmacokinetic clearance.

Within each latent subgroup, a positive association between the covariate and individual clearance deviations was simulated. However, the two subgroups were characterized by markedly different covariate distributions and baseline clearance levels, such that aggregation across groups resulted in a weak, null, or reversed marginal correlation between the covariate and clearance. This design mimics real-world pharmacometric situations in which unmeasured factors such as disease severity, treatment protocol, or center effects act as confounders.

Importantly, the latent grouping variable was not used in the primary analyses. Instead, the scenario focused on assessing whether Wasserstein-based distributional comparisons could reveal residual structure incompatible with a simple marginal association. Empirical Bayes estimates of clearance deviations were compared across covariate strata using permutation-based Wasserstein distance testing, despite the absence of an explicit grouping variable in the analysis.

This scenario was intended to evaluate the ability of Wasserstein distance to detect aggregation-induced misspecification, highlighting situations in which classical correlation or regression analyses may provide misleading conclusions.

Simulated ETA values were generated directly rather than estimated from a full NLME model. These simulations were designed to illustrate statistical behavior under controlled conditions rather than to reproduce realistic NLME estimation settings. In particular, they do not account for shrinkage effects, which may substantially affect the applicability of the proposed approach in practice.

#### 2.4.8 Statistical Comparisons

Analyses were performed using grouped covariates (binary or quartiles), while continuous relationships were explored using regression approaches. For both simulation scenarios, the performance of Wasserstein-based methods was compared to classical approaches between the simulated covariate and clearance deviations:

- Scenario A (threshold): Pearson correlation, Student’s t-test, linear regression, and Wasserstein permutation testing.
- Scenario B (U-shaped): Pearson correlation, ANOVA, quadratic regression, and Wasserstein pairwise permutation testing.
- Scenario C (Simpson’s paradox): Pearson correlation, linear regression and Wasserstein permutation testing. Wasserstein-based analyses were performed by comparing distributions of clearance deviations across covariate strata defined without reference to the latent grouping variable.

Permutation testing (10,000 iterations) was used to assess the statistical significance of observed Wasserstein distances, evaluating whether residual distributional heterogeneity persisted despite weak or misleading marginal associations. All permutation tests were based on 10,000 iterations. Statistical significance was defined as p < 0.05.

#### 2.4.9 Software and implementation

All analyses were conducted using R version 4.3.0 (R Foundation for Statistical Computing, Vienna, Austria). Wasserstein distances were computed using the *transport* package (version 0.14-7). Classical statistical analyses employed base R functions (*t.test*, *aov*, *lm*). Data visualization was performed using *ggplot2* (version 3.4.2) and *patchwork* (version 1.1.2). An R script, an accompanying explanatory Word document and the NONMEM control streams corresponding to the models described in the manuscript, which include the full model equations and implementation details are provided as Supplementary Material to illustrate how to perform the analysis.

### 2.5 Data availability

The dataset used in this study is available from the corresponding author upon reasonable request.

## 3 Results

### 3.1 Real data analysis: genetic and physiological covariates

The η-shrinkage for clearance in the FINAL model was 6.98%, below the 30% threshold recommended for reliable ETA-based diagnostics (Savic and Karlsson, 2009), supporting the validity of using empirical Bayes estimates for distributional comparisons across all covariate analyses presented below.

### 3.2 Genetic covariate (CT01) specification assessment

**FIGURE 1:**
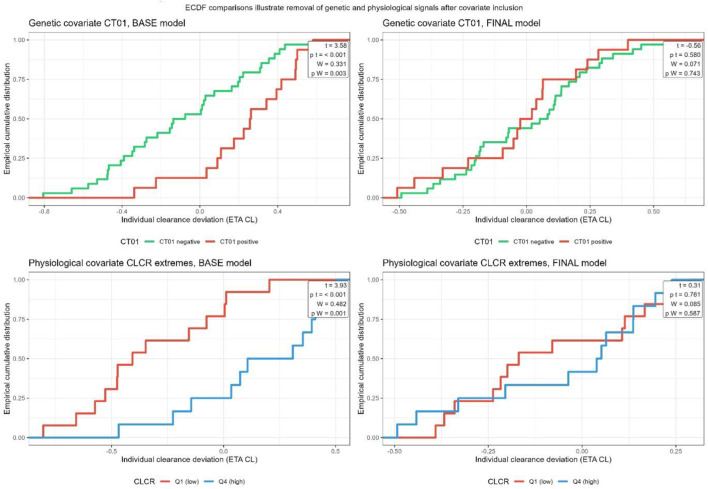
Real data: residual covariate signal in ETA CL for BASE versus FINAL models.

Analysis of the CT01 genetic polymorphism demonstrated adequate covariate specification in the GENET_ALL and FINAL models. In the GENET_ALL model, distributional comparisons showed a reduction in residual differences compared to the BASE model (W = 0.244), although some variability remained. This intermediate result supports partial covariate specification prior to inclusion of creatinine clearance in the FINAL model. Empirical cumulative distribution functions of individual clearance deviations (ETA_CL), stratified by CT01 status and by creatinine clearance strata, are shown for the BASE and FINAL models (Figure 1). The distribution of ETA_CL values showed no significant differences between CT01-positive patients (CT or TT genotypes, n = 16) and CT01-negative patients (CC genotype, n = 34) in the FINAL model:

- Classical t-test: p = 0.580 (no significant difference).
- Wasserstein permutation test: W = 0.071, p = 0.741 (10,000 iterations). These analyses were performed using empirical Bayes estimates derived from the final population pharmacokinetic model and reflect remaining interindividual variability after covariate inclusion.
- Interpretation: The absence of significant distributional differences confirms that the genetic covariate effect is adequately captured by the model, leaving no residual correlation between CT01 status and unexplained clearance variability.

### 3.3 Creatinine clearance (CLCR) specification assessment

Patients were stratified into four quartiles based on creatinine clearance values (Q1: < 71.4 mL/min, Q2: 73.4-90.2 mL/min, Q3: 90.2-116.4 mL/min, Q4: > 116.4 mL/min). In the FINAL model, ETA_CL distributions showed no significant differences across CLCR quartiles:

- ANOVA: F (3, 46) = 1.37, p = 0.264
- Linear regression (ETA_CL ∼ CLCR): R^2^ = 0.001, p = 0.851
- Wasserstein pairwise tests (all comparisons): p > 0.05 after Bonferroni correction

These results confirm adequate specification of the creatinine clearance covariate in the FINAL model. The absence of residual distributional differences across CLCR strata is visually illustrated in Figure 1. The overlapping ETA_CL distributions across CLCR quartiles indicate that renal function effects are adequately captured by the model equations.

### 3.4 Reduction in distributional discrepancy quantified by R^2^ wasserstein

**FIGURE 2:**
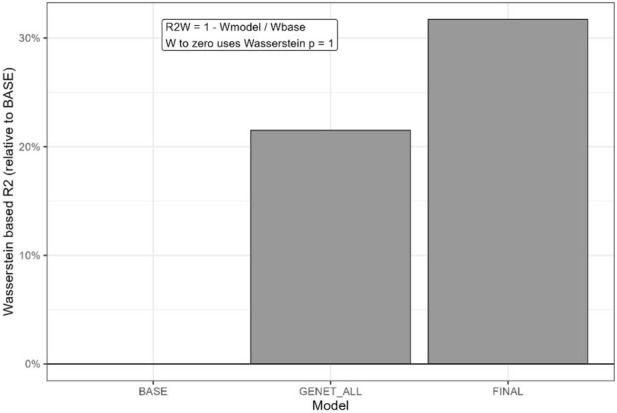
Wasserstein based R2 across nested models.

The Wasserstein-based R^2^ metric quantified the progressive reduction in unexplained distributional discrepancy across nested models (Table 1; Figure 2). The Wasserstein distances reported correspond to W_to_zero, defined as the distance between the empirical distribution of ETA_CL and a reference distribution centered at zero, and therefore do not rely on covariate grouping.

**TABLE 1:** Wasserstein-based R^2^ quantifying the progressive reduction in distributional discrepancy across the nested population pharmacokinetic models.

Model	Wasserstein distance to zero (W_to_zero, ETA_CL)	R^2^ Wasserstein
BASE (no covariates)	0.310	—
GENET_ALL (CT01)	0.244	0.215
FINAL (CT01 + CLCR)	0.212	0.317

W_to_zero is the first‐order Wasserstein distance between the empirical distribution of ETA_CL and the reference (zero) distribution; R^2^ Wasserstein is computed relative to the BASE model. CT01, genetic covariate; CLCR, creatinine clearance.

This progressive reduction in distributional discrepancy across the BASE, GENET_ALL, and FINAL models is summarized in Figure 2. The FINAL model demonstrated an R^2^_W of 0.317, indicating that 31.7% of the baseline distributional discrepancy was reduced by the combination of genetic and renal function covariates. The sequential addition of covariates (BASE → GENET_ALL → FINAL) showed progressive improvement in reduction in distributional discrepancy.

### 3.5 Simulation studies: detection of non-linear relationships

#### 3.5.1 Scenario A: threshold effect detection

In the threshold effect scenario (albumin < 35 g/L), all methods detected significant differences, although they provided different types of information about the relationship (Table 2A).

**TABLE 2A:** Scenario A (threshold effect, albumin < 35 g/L): comparison of statistical methods for detecting the simulated relationship between albumin and individual clearance deviations (ETA_CL). All methods reached significance, but provide different information about the relationship.

Method	Statistic	P-value
Pearson correlation	r = −0.58	<0.001
t-test (2 groups)	t = 11.19	<0.001
Linear regression	R2 = 0.335	<0.001
Wasserstein distance	W = 0.503	<0.001

**FIGURE 3:**
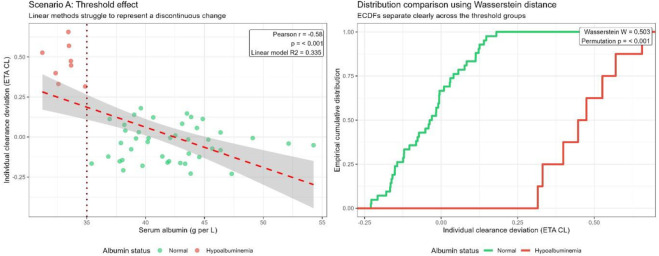
Simulation Scenario A: Threshold effect of albumin on clearance.

The simulated threshold relationship between albumin concentration and individual clearance deviations, together with distributional comparisons across albumin groups, is illustrated in Figure 3. All methods detected significant differences in this threshold scenario. The grouping based on the hypoalbuminemia cutoff (35 g/L) directly reflects the simulated threshold, which facilitates detection by group-based approaches such as the t-test. Linear regression showed a poorer fit (R^2^ = 0.335), reflecting its inability to capture a discontinuous relationship. In this context, the Wasserstein distance provides a complementary perspective by quantifying the global distributional shift between groups, rather than offering a clear advantage over classical methods when the threshold is explicitly defined.

#### 3.5.2 Scenario B: U-shaped relationship detection

The U-shaped relationship presented a scenario in which Pearson correlation did not detect the relationship, whereas ANOVA and quadratic regression identified significant differences (Table 2B). These methods, however, rely on different assumptions or levels of prior model specification.

**TABLE 2B:** Scenario B (U-shaped relationship): comparison of statistical methods. Pearson correlation fails to detect the association, whereas ANOVA, quadratic regression, and the Wasserstein distance (computed separately for the Hypo–Normal and Hyper–Normal subgroups) identify significant differences.

Method	Statistic	P-value
Pearson correlation	r = −0.19	0.181
ANOVA (3 groups)	F = 44.99	<0.001
Quadratic regression	R^2^ = 0.479	<0.001
Wasserstein (Hypo- Normal)	W = 0.428	<0.001
Wasserstein (Hyper-Normal)	W = 0.309	<0.001

The Wasserstein-based approach also identified distributional differences across groups without requiring explicit model specification, providing a complementary tool for exploratory analysis rather than a superior alternative.

**FIGURE 4:**
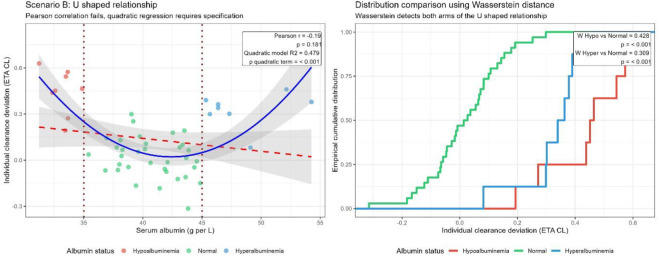
Simulation Scenario B: U shaped effect of albumin on clearance.

Wasserstein distance systematically detected both arms of the U-shape without model specification: hypoalbuminemia effect (W = 0.428, p < 0.001) and hyperalbuminemia effect (W = 0.309, p < 0.001). These two distributional deviations are visually captured by the ECDF comparisons in Figure 4. In addition to the pairwise comparisons, a global Wasserstein index defined as the mean of the three pairwise distances across the hypo, normal, and hyper groups was W = 0.288, supporting an overall distributional departure consistent with a U-shaped relationship.

It should be noted that the interpretation of these results depends on the chosen stratification of the covariate. Alternative groupings, such as quartiles or different cutoffs, could influence the sensitivity of both classical and Wasserstein-based analyses.

#### 3.5.3 Scenario C: Simpson’s paradox–like aggregation effect

In the Simpson’s paradox–like scenario, marginal association analyses yielded potentially misleading results (Table 2C).

**TABLE 2C:** Scenario C (Simpson’s paradox–like aggregation effect): comparison of statistical methods. Marginal analyses (Pearson correlation, linear regression) suggest a strong global association that is misleading once subgroup structure is accounted for; the Wasserstein distance (Q1 vs Q4) characterizes the distributional difference.

Method	Statistic	P-value
Pearson correlation	r = −0.68	<0.001
Linear regression	R^2^ = 0.464	<0.001
Wasserstein (Q1 vs. Q4)	W = 0.572	<0.001

Pearson correlation indicated a strong global association between albumin and clearance deviations (r = −0.68, p < 0.001), and linear regression suggested a substantial proportion of explained variance (R^2^ = 0.464, p < 0.001). However, these marginal analyses reflected aggregation across heterogeneous subpopulations rather than a homogeneous covariate effect.

**FIGURE 5:**
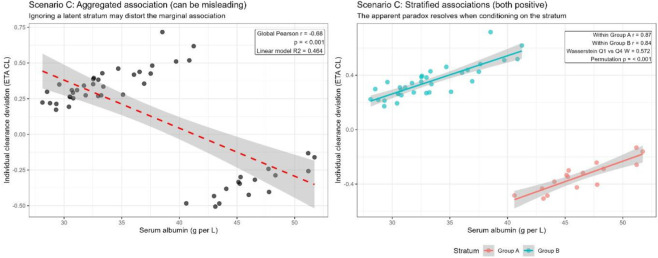
Simulation Scenario C: Simpson’s paradox–like aggregation effect.

In contrast, Wasserstein-based distributional analysis revealed marked heterogeneity in individual clearance deviations across albumin strata. Comparison between the lowest and highest albumin quartiles (Q1 vs. Q4) showed a large Wasserstein distance (W = 0.572, p < 0.001), indicating substantial distributional separation despite the apparent adequacy of the global regression model. This discrepancy is visually illustrated in Figure 5, where aggregation obscures opposing within-stratum trends.

In addition to pairwise comparisons, a global Wasserstein index computed as the mean of the distributional distances across albumin strata was Wmean = 0.383, supporting the presence of residual structure incompatible with a single marginal association.

## 4 Discussion

### 4.1 Principal findings

This tutorial demonstrated the application of Wasserstein distance as a complementary tool for population pharmacokinetic model validation, with particular advantages for detecting non-linear covariate relationships. Our analyses revealed three key findings:

- Adequate covariate specification in real MTX data: Both genetic (CT01) and physiological (CLCR) covariates showed no residual correlation with unexplained clearance variability in the FINAL model, confirming proper model specification (Simon et al., 2013). This absence of residual covariate-related distributional signal is illustrated in Figures 1, 2. The Wasserstein-based R^2^ metric quantified 31.7% reduction in distributional discrepancy from the BASE model (R^2^_W = 0.317 for the FINAL model).
- Complementary insights for threshold effects: In this threshold scenario, all methods detected group differences, while linear regression showed a poorer fit (R^2^ = 0.335) because it does not capture a discontinuous relationship well. Because the analysis used the same hypoalbuminemia cutoff as the one defining the simulated threshold, detection was facilitated for group-based approaches. In this context, the Wasserstein distance provided a complementary distribution-based perspective by quantifying the global shift between groups. This threshold scenario is illustrated in the simulation results shown in Figure 3.
- Relevance for complex relationships: The U-shaped albumin-clearance relationship was not detected by Pearson correlation (r = −0.19, p = 0.181), whereas ANOVA and quadratic regression identified significant differences. This complex nonmonotonic relationship is illustrated in Figure 4. In this setting, the Wasserstein-based approach also detected distributional differences across groups without requiring explicit specification of a parametric functional form, supporting its usefulness as a complementary tool for exploratory covariate analysis (Mould and Upton, 2012; Bauer, 2019; Byon et al., 2013).
- Detection of aggregation-induced misspecification (Simpson’s paradox–like scenario): A third simulation scenario highlighted situations in which marginal association measures are misleading due to aggregation over latent subgroups. In this Simpson’s paradox–like setting, classical correlation and linear regression suggested a strong global association between albumin and clearance deviations, despite the absence of a homogeneous covariate effect across individuals. In contrast, Wasserstein-based distributional analysis revealed substantial residual heterogeneity across albumin strata, exposing aggregation-induced misspecification that remained invisible to marginal methods. This scenario, illustrated in Figure 5, emphasizes the ability of Wasserstein distance to detect violations of conditional homogeneity even when standard regression diagnostics appear satisfactory.

### 4.2 Complementary value relative to classical methods

Wasserstein distance offers several methodological advantages compared to classical pharmacometric approaches.

### 4.3 Model-Free detection

Classical methods such as correlation and linear regression are tied to specific assumptions about the form of the relationship between covariates and individual deviations. Pearson correlation assumes linearity, polynomial regression requires specifying the degree, and threshold-based analyses require defining cutoff values. In contrast, Wasserstein distance compares entire distributions without requiring specification of a parametric functional form, which may be useful for exploratory analysis when the covariate effect shape is uncertain (Ribbing and Jonsson, 2004; Byon et al., 2013). This property is illustrated by the threshold and U-shaped simulation scenarios (Figures 3, 4). However, as shown in these simulations, the interpretation of distributional differences may depend on the chosen covariate stratification, and alternative grouping strategies, such as different cutoffs or quantiles, may influence the sensitivity of the analysis. The Simpson’s paradox–like simulation further illustrates that Wasserstein distance can reveal aggregation-induced misspecification in situations where marginal correlation or regression suggests an apparently adequate covariate effect (Figure 5).

### 4.4 Global distributional assessment

Traditional tests focus on specific distribution moments (t-test compares means, F-test compares variances) or maximum deviations (Kolmogorov-Smirnov test). Wasserstein distance integrates differences across the entire cumulative distribution, capturing location, dispersion, skewness, and tail behavior simultaneously (Villani, 2008; Peyré and Cuturi, 2019; Kolouri et al., 2017; Givens and Shortt, 1984). This global assessment is particularly valuable when covariate effects manifest differently across the ETA distribution (e.g., affecting high clearance patients more than low clearance patients).

### 4.5 Robustness to outliers and non-normality

Population pharmacokinetic analyses frequently encounter skewed ETA distributions, particularly for clearance parameters constrained to positive values. Wasserstein distance does not assume normality and shows robustness to outliers due to its L1-based formulation, compared to classical methods based on squared deviations (correlation, regression) that are sensitive to extreme values (Savic and Karlsson, 2009; Villani, 2008; Peyré and Cuturi, 2019; Kolouri et al., 2017).

### 4.6 Quantification of model improvement

The R^2^ Wasserstein metric provides an intuitive measure of reduction in distributional discrepancy when adding covariates, analogous to classical R^2^ but based on distributional distances rather than variance decomposition. This facilitates comparison across nested models and quantifies the practical benefit of including additional covariates beyond statistical significance testing (Ribbing and Jonsson, 2004; Taylor et al., 2023). This metric is conceptually related to the classical reduction in random effect standard deviation but extends beyond second-moment summaries by capturing global distributional differences.

### 4.7 Clinical and methodological implications

The findings have several implications for pharmacometric practice and model-informed precision dosing.

### 4.8 Validation of covariate specification

Confirming adequate covariate specification is critical for model-informed precision dosing. Our results demonstrate that proper covariate models eliminate residual correlation between patient characteristics and unexplained variability. This validation ensures that Bayesian forecasting will not systematically over- or under-predict drug exposure in specific patient subgroups, improving dosing accuracy for personalized medicine applications (Darwich et al., 2021).

### 4.9 Discovery of complex covariate effects

The simulation studies highlight clinically plausible scenarios in which classical methods may be insufficient or require stronger model assumptions. Threshold effects occur when physiological parameters cross critical values (e.g., renal function below which clearance becomes impaired). U-shaped relationships exist for numerous covariates (e.g., age effects on enzyme activity, therapeutic drug monitoring with both low and high albumin affecting pharmacokinetics) (Mould and Upton, 2012; Ribbing and Jonsson, 2004). In such settings, Wasserstein-based permutation testing may serve as a complementary screening tool for identifying complex distributional effects during covariate model building.

### 4.10 Integration with existing workflows

Wasserstein distance complements rather than replaces classical pharmacometric tools. We recommend its use alongside traditional diagnostics: (1) use ANOVA or regression for initial covariate screening and effect quantification, (2) apply Wasserstein permutation tests as an additional distribution-based diagnostic to assess residual covariate-related heterogeneity, (3) calculate R^2^ Wasserstein to quantify reduction in distributional discrepancy across nested models, and (4) employ visual predictive checks and goodness-of-fit plots for overall model assessment (Byon et al., 2013; Taylor et al., 2023). This multi-faceted approach leverages the strengths and limitations of each method.

### 4.11 Limitations and considerations

Several limitations merit consideration when applying Wasserstein distance to pharmacometric model validation.

### 4.12 Comparative rather than absolute assessment

Unlike classical hypothesis tests with established significance thresholds, Wasserstein distance values lack universal interpretation criteria. Assessment is primarily comparative (smaller distances indicate better covariate specification) rather than absolute (no consensus on ‘acceptable’ distance values). Importantly, Wasserstein-based model comparisons are parameter-specific and should not be interpreted as global measures of overall model adequacy or predictive performance. In aggregation prone situations such as Simpson’s paradox-like structures, Wasserstein distance does not identify the latent confounder itself but signals the inadequacy of marginal model interpretation through persistent distributional discrepancies. Permutation testing provides p-values for significance, but the magnitude of W requires context-specific interpretation (Villani, 2008; Peyré and Cuturi, 2019). We recommend establishing reference ranges through simulation studies for specific drug-population combinations. In simple scenarios with well-separated group means or predefined thresholds, classical methods such as ANOVA or t-tests may yield similar conclusions. The proposed approach should therefore be considered complementary, particularly in situations involving complex or non-standard distributional differences.

### 4.13 Reliance on empirical bayes estimates

A key limitation of the proposed approach is its reliance on empirical Bayes estimates, which are affected by eta-shrinkage. Reliable interpretation therefore requires sufficiently low shrinkage levels, as high shrinkage may obscure true covariate-parameter relationships (Savic and Karlsson, 2009).

### 4.14 Computational intensity

Permutation-based inference (10,000 iterations in our analyses) requires substantial computation compared to analytical tests. For large datasets or complex covariate structures with multiple testing, computational time may become prohibitive. However, modern computing resources and parallelization make this tractable for most pharmacometric applications. Alternative approaches include bootstrap approximations or analytical asymptotic distributions under development (Peyré and Cuturi, 2019; Kolouri et al., 2017).

### 4.15 Multivariate extension challenges

Our tutorial focused on univariate Wasserstein distances (comparing ETA_CL distributions). Extension to multivariate scenarios (simultaneous assessment of ETA_CL and ETA_V) requires careful consideration of parameter scaling and correlation structure. Multivariate Wasserstein distances exist but require higher-dimensional transportation algorithms with increased computational complexity. For models with multiple random effects, we recommend sequential univariate assessments or dimensionality reduction before applying Wasserstein metrics (Peyré and Cuturi, 2019; Givens and Shortt, 1984).

### 4.16 Sensitivity to sample size

Like all statistical tests, Wasserstein permutation tests may exhibit sensitivity to sample size (Ernst, 2004). Small subgroup sizes may reduce statistical power, particularly in the context of rare or unbalanced covariate categories, consistent with pharmacometric studies showing increased bias and reduced power in small datasets (Mould and Upton, 2013). Bootstrap confidence intervals can help quantify estimation uncertainty, but adequate sample sizes remain important for reliable inference, as emphasized in statistical literature on permutation-based inference (Ernst, 2004) and in pharmacometric studies on covariate model performance (Ribbing and Jonsson, 2004).

### 4.17 Future directions

Several promising research directions emerge from this work.

### 4.18 Automated covariate discovery

Machine learning algorithms could combine Wasserstein-based covariate screening with automated model building. Rather than manually testing hundreds of potential covariates, algorithms could systematically identify distributional imbalances in residual variability, suggest non-linear transformations (thresholds, splines, polynomials), and rank covariates by reduction in distributional discrepancy potential. This would streamline the traditionally labor-intensive covariate model building process (Peyré and Cuturi, 2019; Kolouri et al., 2017; Schiebinger et al., 2019).

### 4.19 Integration with bayesian estimation

Bayesian pharmacometric workflows estimate posterior distributions of population parameters and individual random effects. Wasserstein distances could naturally extend to compare full posterior distributions rather than point estimates, providing uncertainty quantification for covariate effects. Posterior predictive checks using Wasserstein metrics could complement traditional visual predictive checks with distributional discrepancy measures.

### 4.20 External validation metrics

Model validation in external populations requires assessing whether covariate relationships generalize. Wasserstein distances between ETA distributions in development versus validation cohorts could identify population-specific covariate effects that limit model transportability. Large distances would flag when model refinement or local recalibration is needed before clinical implementation.

### 4.21 Regulatory applications

As model-informed drug development gains regulatory acceptance, standardized validation metrics become increasingly important. Wasserstein-based assessments could supplement traditional model qualification procedures by providing objective, quantifiable evidence of adequate covariate specification. This may be particularly valuable for complex covariate models (e.g., pediatric maturation functions, disease progression effects) where graphical diagnostics alone may be insufficient (Darwich et al., 2021; Taylor et al., 2023).

### 4.22 Implications for translational pharmacology

Beyond classical pharmacometric applications, distribution-based approaches such as the Wasserstein distance may also integrate with emerging translational and diagnostic technologies generating complex biological data. For instance, advanced biomimetic systems for tissue repair (Yao et al., 2026), novel receptor-level therapeutic targets (Zhong et al., 2025), or electrochemical probes capturing mitochondrial stress (Hao et al., 2026) produce high-dimensional and heterogeneous biomarker profiles. In such contexts, Wasserstein-based methods could help validate how these biomarkers are incorporated into PK/PD models, ensuring that residual variability is not driven by unaccounted physiological states.

## 5 Conclusion

Wasserstein distance offers a complementary distribution-based approach with clearly defined assumptions, including the requirement for low eta-shrinkage when using empirical Bayes estimates in population pharmacokinetic model validation. Our tutorial demonstrated that this metric can detects both linear and complex non-linear covariate relationships that may be missed by classical methods. The combination of permutation-based hypothesis testing, R^2^

Wasserstein for variance quantification, and visual ECDF comparisons provides a comprehensive toolkit for pharmacometricians. Importantly, this approach is parameter-specific and is intended to quantify the remaining unexplained variability in the corresponding random effect (ETA), rather than overall model fit or predictive performance.

As precision medicine advances, ensuring that population models adequately capture patient-specific factors becomes increasingly critical for dosing accuracy. Wasserstein-based validation helps confirm that models are ready for clinical application, improving confidence in model-informed precision dosing. We recommend integrating Wasserstein distance into standard pharmacometric workflows as a complementary diagnostic alongside traditional methods, particularly when exploring novel covariates or validating complex covariate models.

Supplementary Material includes an example R script and an accompanying explanatory document illustrating the application of the proposed methods.

Empirical cumulative distribution functions of individual clearance deviations (ETA CL) are shown for the BASE and FINAL models. The top row contrasts ETA CL distributions by CT01 status, and the bottom row contrasts extreme CLCR strata, defined as the lowest versus highest quartiles. Each panel reports a two-group t-test and a permutation-based Wasserstein test. A distributional separation is typically observed under BASE that is attenuated or removed under FINAL, supporting appropriate covariate specification.

Model-level improvement is quantified using a Wasserstein-based coefficient of determination defined as

$$R^{2}\_W=1\;‐\;\left(W\_\text{to}\_\text{zero}\left[\text{model}\right]/\;W\_\text{to}\_\text{zero}\left[\text{BASE}\right]\right),$$

where W_to_zero represents the first-order Wasserstein distance between the empirical distribution of individual clearance deviations (ETA_CL) and a reference distribution centered at zero. This metric quantifies the proportional reduction in global distributional dispersion achieved by covariate inclusion.

Values shown: BASE (reference, R^2^_W = 0), GENET_ALL including CT01 genetic covariate (R^2^_W = 0.215), and FINAL including both CT01 and creatinine clearance (R^2^_W = 0.317). The progressive increase demonstrates improved model specification with sequential covariate addition.

Simulated data for 50 subjects illustrate a threshold relationship between serum albumin and individual clearance deviation. Left panel shows albumin versus ETA CL with a linear fit, highlighting that linear approaches do not represent a discontinuous change. Right panel compares ECDFs for subjects below and above the threshold and reports the Wasserstein distance with a permutation-based p value, illustrating a global distributional difference between groups.

Simulated data for 50 subjects illustrate a U-shaped relationship between serum albumin and individual clearance deviation with three albumin strata. Left panel shows albumin versus ETA CL with linear and quadratic fits, illustrating that Pearson correlation does not detect the relationship, while quadratic regression requires explicit model specification. Right panel compares ECDFs across the three groups and reports pairwise Wasserstein distances for low versus normal and high versus normal strata with permutation-based p values, showing consistent detection of both arms of the relationship.

Simulated data for 50 subjects illustrate a Simpson’s paradox–like situation arising from aggregation over latent subgroups. Left panel shows the marginal relationship between serum albumin and individual clearance deviation (ETA CL), suggesting a strong global association based on correlation and linear regression. Right panel compares empirical cumulative distribution functions of ETA CL across extreme albumin strata (lowest versus highest quartiles) and reports the Wasserstein distance with a permutation-based p value. Despite the apparent adequacy of the marginal regression, the distributional comparison reveals substantial heterogeneity in clearance deviations, indicating aggregation induced misspecification of the covariate effect.

## Data Availability

The data analyzed in this study is subject to the following licenses/restrictions: no restriction. Requests to access these datasets should be directed to jean-sebastien.hulot@aphp.fr.
