# Supplementary material for: Distribution-based covariate assessment using wasserstein distance in population pharmacokinetic models
Source: Front Pharmacol. 2026 Jul 6;17:1804989. doi: 10.3389/fphar.2026.1804989 (PMC13382220; doi:10.3389/fphar.2026.1804989)
Supplement: Supplementary file 1 [file DataSheet2.pdf]

Supplementary material

Distribution-Based Covariate Assessment Using Wasserstein Distance in Population Pharmacokinetic Models.

Nicolas SIMON[1], Jean-Sebastien HULOT[2,3], Katharina von FABECK[1]

Affiliation

1 Department of Clinical Pharmacology, APHM, Institut de Neurosciences de la Timone, UMR7289, CNRS, Hôpital Sainte Marguerite, CAP-TV, Aix Marseille University, Marseille, France

2 Université Paris Cité, INSERM, PARCC, F-75015 Paris, France ;

3 CIC1418, AP-HP, Hôpital Européen Georges-Pompidou, F-75015, Paris, France;

Corresponding author:

Nicolas SIMON

Service de Pharmacologie Clinique, Centre Anti-Poison

Hôpital Sainte Marguerite

270 Bd Sainte Marguerite

13009 Marseille

France

Nicolas.simon@ap-hm.fr

1. Explanation of the R code

This R script implements a Wasserstein-based validation workflow to compare two population pharmacokinetic models using individual random effects extracted from NONMEM .phi files.

First, the script loads the required libraries for data manipulation (tidyverse), Wasserstein distance computation (transport), figure assembly (patchwork), and axis formatting (scales). A random seed is set to ensure reproducibility of permutation tests.

The user specifies three input files: a covariate table containing patient identifiers and covariates (ID, CLCR, CT01), and two .phi files corresponding to two competing models, typically a BASE and a FINAL model. The number of permutations used for Wasserstein hypothesis testing is also defined.

Several helper functions are then defined.

A dedicated function reads NONMEM .phi files and extracts individual clearance deviations (ETA CL). Another function formats p-values for consistent display. A core function computes the one-dimensional Wasserstein distance between two groups and assesses its significance using permutation testing. Finally, a function computes the Wasserstein distance between a distribution and zero, which is later used to define a Wasserstein-based R^2.

Covariates are then loaded and cleaned. Creatinine clearance (CLCR) is discretized into quartiles at the patient level, allowing identification of extreme subgroups (lowest and highest quartiles).

Individual ETAs from both models are merged with the covariate table. Model labels are preserved, and covariates are recoded as factors to facilitate stratified analyses.

For each model separately, two types of comparisons are performed. First, genetic status (CT01 positive vs negative) is evaluated using a classical two-sample t-test and a permutation-based Wasserstein test.

Second, physiological extremes of renal function (CLCR Q1 vs Q4) are analyzed using the same dual approach.

The results of these tests are printed to the console and exported as CSV files for direct inclusion in the manuscript.

The script then generates ECDF plots for each covariate and each model. For every panel, both classical statistics (t-test) and Wasserstein statistics are annotated directly on the figure, allowing visual comparison of distributional separation.

Finally, a Wasserstein-based R^2 is computed. For each model, the Wasserstein distance between the ETA distribution and zero is calculated. The BASE model is used as a reference, and the relative reduction in distance for the FINAL model is interpreted as a distributional analogue of variance explained. The corresponding bar plot is generated and saved.

2. Interpretation of numerical results

CT01 results

BASE model

The t-test and Wasserstein test are both highly significant. This indicates a strong residual genetic signal in ETA CL. The distributions of clearance deviations differ substantially between CT01-positive and CT01-negative patients.

FINAL model

Neither the t-test nor the Wasserstein test is significant. The very small Wasserstein distance confirms that the residual distributions overlap almost completely. This indicates that the genetic covariate has been adequately incorporated into the model.

CLCR extremes results

BASE model

Both tests are highly significant, with a large Wasserstein distance. This indicates a strong residual physiological signal, meaning that renal function extremes are still associated with unexplained clearance variability.

FINAL model

Both tests are non-significant, and the Wasserstein distance is small. This shows that the FINAL model successfully removes the residual CLCR-related structure in ETA CL.

Wasserstein-based R^2

The Wasserstein distance to zero decreases from 0.310 in the BASE model to 0.212 in the FINAL model. This corresponds to a Wasserstein-based R^2 of approximately 32 percent. In other words, about one third of the residual distributional dispersion in ETA CL is removed by the FINAL model relative to the BASE model.

3. Interpretation of Figure 1

Figure 1. Real data: residual covariate signal in ETA CL for BASE versus FINAL models

ECDF comparisons illustrate attenuation or removal of residual covariate related distributional signal

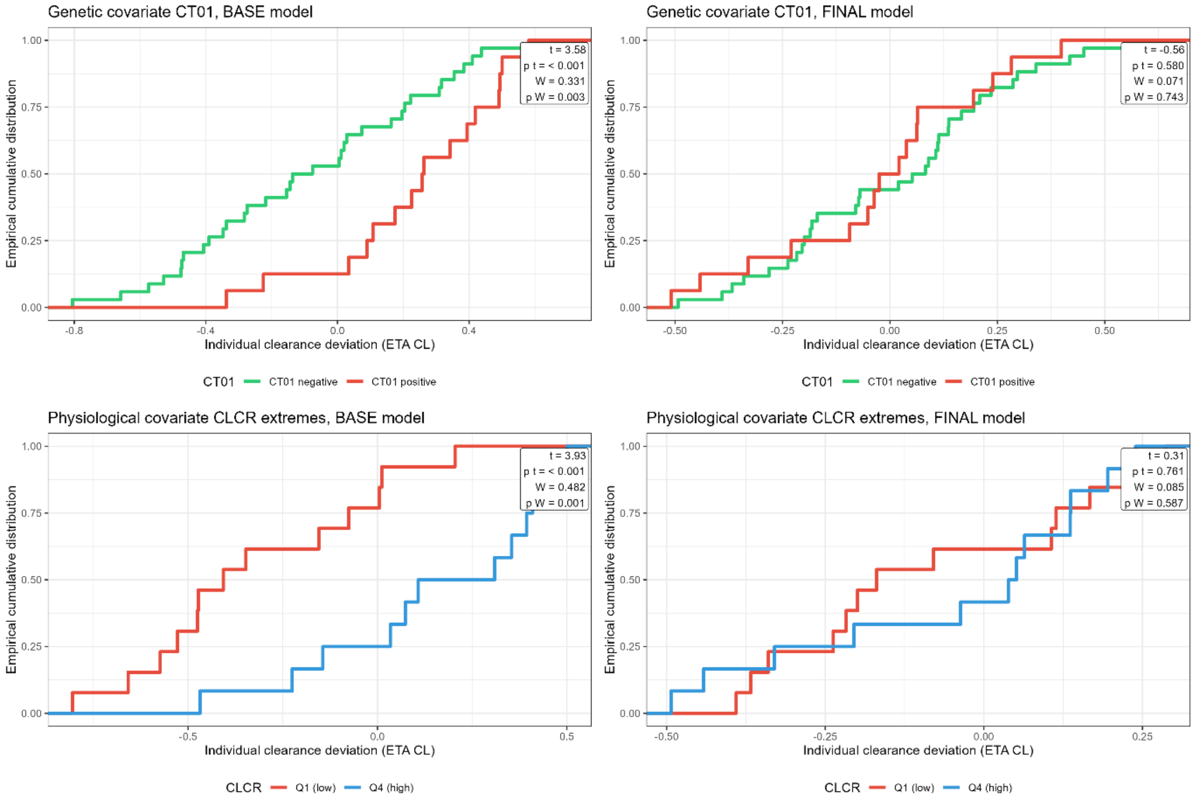

Figure 1 shows empirical cumulative distribution functions of individual clearance deviations stratified by genetic and physiological covariates.

In the BASE model, clear distributional separations are observed for both CT01 and CLCR extremes, reflected by large Wasserstein distances and significant permutation p-values. These patterns indicate residual covariate-related structure in unexplained variability.

In the FINAL model, the ECDF curves largely overlap for both covariates. The corresponding Wasserstein distances are small and non-significant, demonstrating that the FINAL model successfully attenuates or removes residual covariate signals in ETA CL.

4. Interpretation of Figure 2

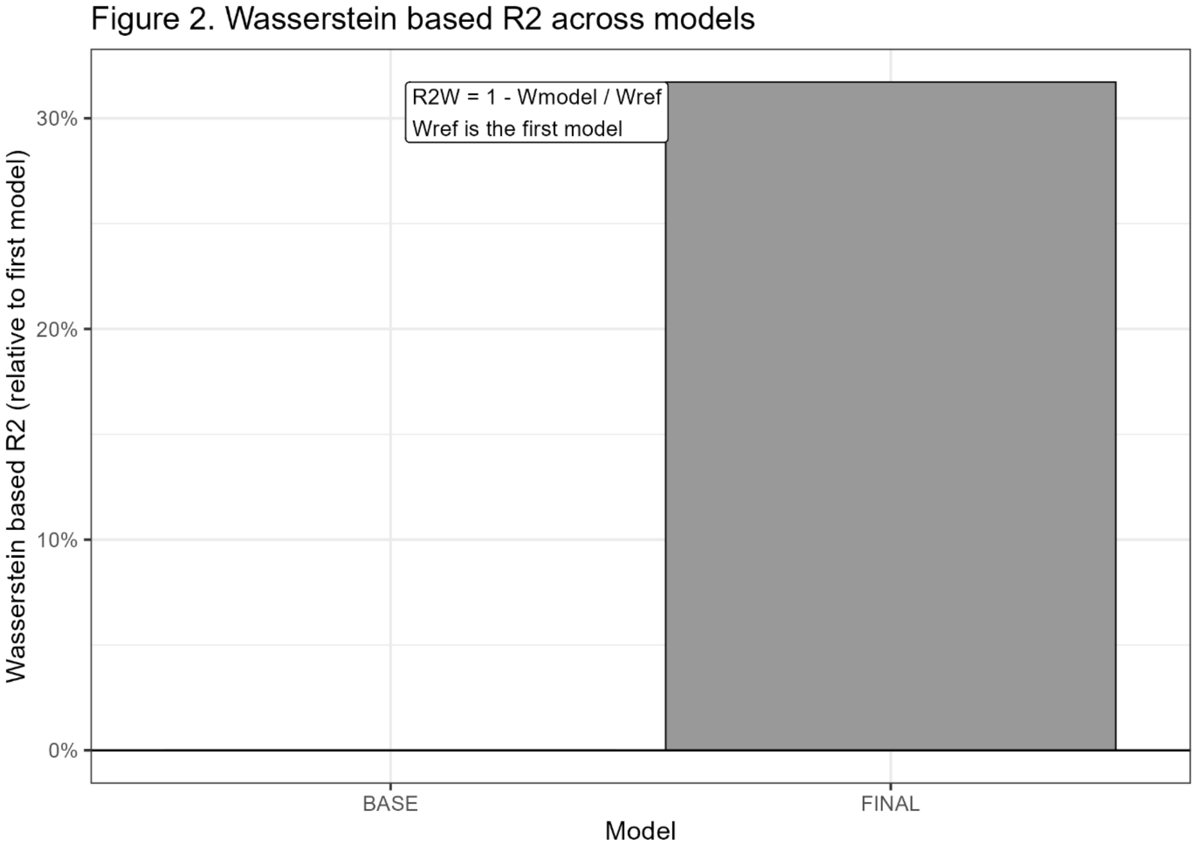

Figure 2 summarizes model-level improvement using a Wasserstein-based R^2. The BASE model defines the reference level of residual distributional dispersion. The FINAL model shows a substantial reduction in Wasserstein distance to zero, corresponding to approximately 32 percent relative improvement. This metric provides a global, distribution-based quantification of model refinement.

Supplementary R Script

```
#=====================================================================
==========
# Supplementary R script
# Wasserstein distance based validation from two NONMEM .phi files
# Inputs:
#   - covariates CSV with columns: ID, CLCR, CT01
#   - two NONMEM .phi files (e.g., BASE and FINAL) containing ID and ETA(1)
# Outputs:
#   - Figure_1_RealData_Covariates_English.png
#   - Figure_2_R2W_English.png
#   - CSV result tables for manuscript integration
#=====================================================================
==========

suppressPackageStartupMessages({
  library(tidyverse)
  library(transport)
  library(patchwork)
  library(scales)
})

#______________________________
# User configuration ----
#______________________________
set.seed(2025)

# Path to covariates file
path_cov <- "mt1_covariates.csv"

# Paths to two .phi files
path_phi_1 <- "base.phi"   # model 1, usually BASE
```

```r
path_phi_2 <- "final.phi"  # model 2, usually FINAL

# Labels used in plots and tables
label_phi_1 <- "BASE"
label_phi_2 <- "FINAL"

# Permutation settings
n_perm <- 10000

#______________________________
# Helpers ----
#______________________________
read_phi <- function(path) {
  # NONMEM .phi are often space separated and include a first line header
  readr::read_table(path, skip = 1, col_types = cols(.default = col_guess()))
}

get_eta_cl <- function(phi_df) {
  stopifnot(all(c("ID", "ETA(1)") %in% names(phi_df)))
  phi_df %>%
    transmute(
      ID = as.integer(ID),
      ETA_CL = as.numeric(`ETA(1)`)
    )
}

fmt_p <- function(p) {
  if (is.na(p)) return("NA")
  if (p < 0.001) return("< 0.001")
  formatC(p, format = "f", digits = 3)
}
```

```
wass_perm_2groups <- function(x, y, n_perm = 10000, seed = 1) {
  set.seed(seed)
  x <- as.numeric(x); y <- as.numeric(y)
  x <- x[is.finite(x)]; y <- y[is.finite(y)]
  stopifnot(length(x) >= 2, length(y) >= 2)

  W_obs <- transport::wasserstein1d(x, y, p = 1)

  all <- c(x, y)
  n_x <- length(x)

  W_perm <- replicate(n_perm, {
    s <- sample(all, replace = FALSE)
    transport::wasserstein1d(s[1:n_x], s[(n_x + 1):length(all)], p = 1)
  })

  p_value <- (sum(W_perm >= W_obs) + 1) / (n_perm + 1)

  list(W_obs = W_obs, p_value = p_value, W_perm = W_perm)
}

W_to_zero <- function(x) {
  x <- as.numeric(x)
  x <- x[is.finite(x)]
  transport::wasserstein1d(x, rep(0, length(x)), p = 1)
}

#______________________________
# Load covariates ----
#______________________________
cov <- readr::read_csv(path_cov, show_col_types = FALSE)
names(cov) <- gsub("^X\\.", "", names(cov))
```

```r
stopifnot(all(c("ID", "CLCR", "CT01") %in% names(cov)))

cov <- cov %>%
  transmute(
    ID   = as.integer(ID),
    CLCR = as.numeric(CLCR),
    CT01 = as.integer(CT01)
  ) %>%
  distinct(ID, .keep_all = TRUE)

cat(sprintf("Patients in covariates file: %d\n", nrow(cov)))

# Define CLCR quartiles once at patient level
cov_q <- cov %>%
  mutate(CLCR_Q = ntile(CLCR, 4)) %>%
  mutate(
    CLCR_Q = factor(
      CLCR_Q,
      levels = 1:4,
      labels = c("Q1 (low)", "Q2", "Q3", "Q4 (high)")
    )
  ) %>%
  select(ID, CLCR_Q)

#______________________________
# Load two models ETAs and merge
#______________________________
eta_1 <- get_eta_cl(read_phi(path_phi_1)) %>% mutate(Model = label_phi_1)
eta_2 <- get_eta_cl(read_phi(path_phi_2)) %>% mutate(Model = label_phi_2)

eta_all <- bind_rows(eta_1, eta_2) %>%
```

```
  inner_join(cov, by = "ID") %>%
  left_join(cov_q, by = "ID") %>%
  mutate(
    Model = factor(Model, levels = c(label_phi_1, label_phi_2)),
    CT01_f = factor(CT01, levels = c(0, 1), labels = c("CT01 negative", "CT01 positive")),
    CLCR_extreme = case_when(
      CLCR_Q %in% c("Q1 (low)", "Q4 (high)") ~ as.character(CLCR_Q),
      TRUE ~ NA_character_
    ),
    CLCR_extreme = factor(CLCR_extreme, levels = c("Q1 (low)", "Q4 (high)"))
  )

#______________________________
# Tests for Figure 1 style results: CT01 and CLCR extremes ----
#______________________________
models_for_fig1 <- eta_all

ct01_results <- models_for_fig1 %>%
  group_by(Model) %>%
  group_modify(~{
    df <- .x
    model_name <- as.character(.y$Model[1])

    x0 <- df$ETA_CL[df$CT01 == 0]
    x1 <- df$ETA_CL[df$CT01 == 1]

    tt <- t.test(x1, x0)
    ws <- wass_perm_2groups(
      x1, x0,
      n_perm = n_perm,
      seed = ifelse(model_name == label_phi_2, 101, 100)
    )
```

```
    tibble(
      t_stat = as.numeric(tt$statistic),
      p_t    = tt$p.value,
      W      = ws$W_obs,
      p_W    = ws$p_value
    )
  }) %>%
  ungroup()

clcr_results <- models_for_fig1 %>%
  filter(!is.na(CLCR_extreme)) %>%
  group_by(Model) %>%
  group_modify(~{
    df <- .x
    model_name <- as.character(.y$Model[1])

    x_q1 <- df$ETA_CL[df$CLCR_extreme == "Q1 (low)"]
    x_q4 <- df$ETA_CL[df$CLCR_extreme == "Q4 (high)"]

    tt <- t.test(x_q4, x_q1)
    ws <- wass_perm_2groups(
      x_q4, x_q1,
      n_perm = n_perm,
      seed = ifelse(model_name == label_phi_2, 201, 200)
    )

    tibble(
      t_stat = as.numeric(tt$statistic),
      p_t    = tt$p.value,
      W      = ws$W_obs,
      p_W    = ws$p_value
```

```
    )
  }) %>%
  ungroup()

print(ct01_results)

cat("\nCLCR extremes results (Q1 vs Q4):\n")
print(clcr_results)

write.csv(ct01_results, "RealData_CT01_Results_2models.csv", row.names = FALSE)
write.csv(clcr_results, "RealData_CLCR_Extremes_Results_2models.csv", row.names =
FALSE)

#______________________________
# Figure 1 in English: ECDFs for CT01 and CLCR extremes, both models ----
#______________________________
col_ct01 <- c("CT01 negative" = "#2ecc71", "CT01 positive" = "#e74c3c")
col_clcr <- c("Q1 (low)" = "#e74c3c", "Q4 (high)" = "#3498db")

make_ct01_ecdf <- function(model_name) {
  df <- models_for_fig1 %>% filter(Model == model_name)
  ann <- ct01_results %>% filter(Model == model_name) %>% slice(1)

  ggplot(df, aes(x = ETA_CL, color = CT01_f)) +
    stat_ecdf(geom = "step", linewidth = 1.3) +
    scale_color_manual(values = col_ct01) +
    annotate(
      "label",
      x = Inf, y = Inf,
      label = paste0(
        "t = ", sprintf("%.2f", ann$t_stat), "\n",
        "p t = ", fmt_p(ann$p_t), "\n",
        "W = ", sprintf("%.3f", ann$W), "\n",
```

```
      "p W = ", fmt_p(ann$p_W)
    ),
    hjust = 1.05, vjust = 1.25,
    fill = "white", size = 3.2
  ) +
  labs(
    title = paste0("Genetic covariate CT01, ", model_name, " model"),
    x = "Individual clearance deviation (ETA CL)",
    y = "Empirical cumulative distribution",
    color = "CT01"
  ) +
  theme_bw(base_size = 11) +
  theme(legend.position = "bottom")
}

make_clcr_ecdf <- function(model_name) {
 df <- models_for_fig1 %>%
  filter(Model == model_name) %>%
  filter(!is.na(CLCR_extreme))
 ann <- clcr_results %>% filter(Model == model_name) %>% slice(1)

 ggplot(df, aes(x = ETA_CL, color = CLCR_extreme)) +
  stat_ecdf(geom = "step", linewidth = 1.3) +
  scale_color_manual(values = col_clcr) +
  annotate(
    "label",
    x = Inf, y = Inf,
    label = paste0(
      "t = ", sprintf("%.2f", ann$t_stat), "\n",
      "p t = ", fmt_p(ann$p_t), "\n",
      "W = ", sprintf("%.3f", ann$W), "\n",
      "p W = ", fmt_p(ann$p_W)
```

```
      ),
      hjust = 1.05, vjust = 1.25,
      fill = "white", size = 3.2
    ) +
    labs(
      title = paste0("Physiological covariate CLCR extremes, ", model_name, " model"),
      x = "Individual clearance deviation (ETA CL)",
      y = "Empirical cumulative distribution",
      color = "CLCR"
    ) +
    theme_bw(base_size = 11) +
    theme(legend.position = "bottom")
}

fig1_ct01_m1 <- make_ct01_ecdf(label_phi_1)
fig1_ct01_m2 <- make_ct01_ecdf(label_phi_2)
fig1_clcr_m1 <- make_clcr_ecdf(label_phi_1)
fig1_clcr_m2 <- make_clcr_ecdf(label_phi_2)

Figure_suppl_1 <- (fig1_ct01_m1 | fig1_ct01_m2) / (fig1_clcr_m1 | fig1_clcr_m2) +
  plot_annotation(
    title = paste0("Figure 1. Real data: residual covariate signal in ETA CL for ", label_phi_1, "
versus ", label_phi_2, " models"),
    subtitle = "ECDF comparisons illustrate attenuation or removal of residual covariate related
distributional signal",
    theme = theme(
      plot.title = element_text(size = 14, face = "bold", hjust = 0.5),
      plot.subtitle = element_text(size = 11, hjust = 0.5)
    )
  )

Figure_suppl_1
ggsave("Figure_1_RealData_Covariates_suppl.png", Figure_suppl_1,
```

```
       width = 14, height = 10, dpi = 300, bg = "white")

#________________________________
# Figure 2: Wasserstein based R2 across two models (distance to zero) ----
#________________________________
r2_w <- eta_all %>%
  group_by(Model) %>%
  summarise(W_to_zero = W_to_zero(ETA_CL), .groups = "drop") %>%
  mutate(
    W_ref = W_to_zero[Model == label_phi_1][1],
    R2_W  = 1 - (W_to_zero / W_ref)
  ) %>%
  select(Model, W_to_zero, R2_W)

cat("\nWasserstein based R2 (reference is first model):\n")
print(r2_w)

write.csv(r2_w, "RealData_R2W_2models.csv", row.names = FALSE)

Figure_suppl_2 <- ggplot(r2_w, aes(x = Model, y = R2_W)) +
  geom_col(fill = "gray60", color = "black", linewidth = 0.3) +
  geom_hline(yintercept = 0, linewidth = 0.4) +
  scale_y_continuous(labels = percent_format(accuracy = 1)) +
  annotate(
    "label",
    x = 1.03, y = max(r2_w$R2_W, na.rm = TRUE),
    hjust = 0, vjust = 1,
    label = paste0(
      "R2W = 1 - Wmodel / Wref\n",
      "Wref is the first model"
    ),
    fill = "white", size = 3.2
```

```
  ) +
  labs(
    title = "Figure 2. Wasserstein based R2 across models",
    x = "Model",
    y = "Wasserstein based R2 (relative to first model)"
  ) +
  theme_bw(base_size = 11)

Figure_suppl_2
ggsave("Figure_2_R2W_suppl.png", Figure_suppl_2,
       width = 7, height = 5, dpi = 300, bg = "white")
```
