# Supplementary material for: Distribution-based covariate assessment using wasserstein distance in population pharmacokinetic models
Source: Front Pharmacol. 2026 Jul 6;17:1804989. doi: 10.3389/fphar.2026.1804989 (PMC13382220; doi:10.3389/fphar.2026.1804989)
Supplement: Supplementary file 2 [file DataSheet3.pdf]

Control Streams

The following control streams correspond to the four models described in the manuscript: BASE (no covariates), GENET_ALL (CT01 on CL and V1), and FINAL (CT01 and CLCR on CL, CT01 on V1).

BASE MODEL

```
$PROB MTX

$INPUT ID DAT1=DROP TIME AMT RATE DV

EVID OCC=DROP CT12 CT01 EX12 EX10 EX25 CGT=DROP CGA=DROP CAT=DROP TGT=DROP

P12=DROP P26=DROP P21=DROP BW X AGE HT=DROP BSA=DROP SCR CLCR ;PROT ALB IND
VIH CHIM SUB DOXO VINC CYCL AINS CORT IPP


$DATA ..\mt1.csv IGNORE=#

$SUBROUTINES  ADVAN11 TRANS4

$PK


;IF (CT01.EQ.1) THEN ; MUTATED

;FALL=THETA(7)

;ELSE

;FALL=1;WILD

;ENDIF


  CL=THETA(1)*EXP(ETA(1))

  V1=THETA(2)*EXP(ETA(2))

  Q2=THETA(3)

  V2=THETA(4)

  Q3=THETA(5)

  V3=THETA(6)

  S1=V1


IF (EVID.EQ.1.OR.EVID.EQ.4) THEN

 TDOS=TIME
```

```
  TAD=0.0
ENDIF
IF (EVID.EQ.0.OR.EVID.EQ.2) TAD=TIME-TDOS
;IF (EVID.EQ.1) TAD=TIME-TDOS

$THETA   (0,4); CL
$THETA   (0,20);V1
$THETA   (0,.5); Q2
$THETA  (0,5);  V2
$THETA   (0,.1);Q3
$THETA   (0,5);V3
;$THETA (0,.7); GENET
;$THETA (0,.1)

$OMEGA BLOCK(2)
.4    ; OMCL
.04 .4; OMV1

$ERROR
W=F
IPRED = F
IRES=DV-IPRED
IWRES=0
IF (W.GT.0) IWRES=IRES/W
Y=F*EXP(EPS(1))

$SIGMA
1;SIG

$EST METHOD=1 NOABORT INTERACTION MAXEVAL=5000  PRINT=5 POSTHOC
$COV
```

```
$TABLE ID TAD TDOS TIME IPRED IRES IWRES CL V1 CLCR CT01 ONEHEADER NOPRINT
FILE=base.fit

$TABLE ID V1 CL Q2 V2 Q3 V3 CT12 CT01 EX12 EX10 EX25

NOAPPEND NOPRINT FIRSTONLY FILE=param.fit

$TABLE ID TAD TIME DV PRED WRES RES IPRED NPRED NRES

  CPRED CRES CWRES

  EPRED ERES EWRES NPDE

  ESAMPLE=300 SEED=12334 NOAPPEND NOPRINT

  FILE=final.fit
```

GENET ALL MODEL

```
$PROB  MTX

$INPUT ID DAT1=DROP TIME AMT RATE DV

EVID OCC=DROP CT12 CT01 EX12 EX10 EX25 CGT=DROP CGA=DROP CAT=DROP TGT=DROP

P12=DROP P26=DROP P21=DROP BW X AGE HT=DROP BSA=DROP SCR CLCR ;PROT ALB IND
VIH CHIM SUB DOXO VINC CYCL AINS CORT IPP


$DATA ..\mt1.csv IGNORE=#

$SUBROUTINES  ADVAN11 TRANS4

$PK


IF (CT01.EQ.1) THEN ; MUTATED

FALL=THETA(7)

ELSE

FALL=1;WILD

ENDIF


   CL=THETA(1)*FALL*(EXP(ETA(1)))

   V1=THETA(2)*FALL*(EXP(ETA(2)))

   Q2=THETA(3)

   V2=THETA(4)
```

```
  Q3=THETA(5)
  V3=THETA(6)
  S1=V1

IF (EVID.EQ.1.OR.EVID.EQ.4) THEN
 TDOS=TIME
 TAD=0.0
ENDIF
IF (EVID.EQ.0.OR.EVID.EQ.2) TAD=TIME-TDOS
;IF (EVID.EQ.1) TAD=TIME-TDOS


$THETA   (0,4); CL
$THETA   (0,20);V1
$THETA   (0,.5); Q2
$THETA  (0,5);  V2
$THETA   (0,.1);Q3
$THETA   (0,5);V3
$THETA (0,.7); GENET
;$THETA (0,.1)

$OMEGA BLOCK(2)
.4    ; OMCL
.04 .4; OMV1

$ERROR
W=F
IPRED = F
IRES=DV-IPRED
IWRES=0
IF (W.GT.0) IWRES=IRES/W
```

```
Y=F*EXP(EPS(1))

$SIGMA
1;SIG

$EST METHOD=1 NOABORT INTERACTION MAXEVAL=5000  PRINT=5 POSTHOC
$COV
$TABLE ID TAD TDOS TIME IPRED IRES IWRES CL V1 CLCR CT01 ONEHEADER NOPRINT
FILE=genet_all.fit
$TABLE ID V1 CL Q2 V2 Q3 V3 CT12 CT01 EX12 EX10 EX25
NOAPPEND NOPRINT FIRSTONLY FILE=param.fit
$TABLE ID TAD TIME DV PRED WRES RES IPRED NPRED NRES
  CPRED CRES CWRES
  EPRED ERES EWRES NPDE
  ESAMPLE=300 SEED=12334 NOAPPEND NOPRINT
  FILE=final.fit
```

FINAL MODEL

```
$PROB  MTX
$INPUT ID DAT1=DROP TIME AMT RATE DV
EVID OCC=DROP CT12 CT01 EX12 EX10 EX25 CGT=DROP CGA=DROP CAT=DROP TGT=DROP
P12=DROP P26=DROP P21=DROP BW X AGE HT=DROP BSA=DROP SCR CLCR ;PROT ALB IND
VIH CHIM SUB DOXO VINC CYCL AINS CORT IPP

$DATA ..\mt1.csv IGNORE=#
$SUBROUTINES  ADVAN11 TRANS4
$PK

IF (CT01.EQ.1) THEN ; MUTATED
FALL=THETA(7)
ELSE
```

```
FALL=1;WILD
ENDIF

   CL=((THETA(1)*FALL)+(THETA(8)*(CLCR/89)))*(EXP(ETA(1)))
   V1=THETA(2)*FALL*(EXP(ETA(2)))
   Q2=THETA(3)
   V2=THETA(4)
   Q3=THETA(5)
   V3=THETA(6)
   S1=V1

IF (EVID.EQ.1.OR.EVID.EQ.4) THEN
  TDOS=TIME
  TAD=0.0
ENDIF
IF (EVID.EQ.0.OR.EVID.EQ.2) TAD=TIME-TDOS
;IF (EVID.EQ.1) TAD=TIME-TDOS

$THETA   (0,4); CL
$THETA   (0,20);V1
$THETA   (0,.5); Q2
$THETA  (0,5);  V2
$THETA   (0,.1);Q3
$THETA   (0,5);V3
$THETA (0,.7); GENET
$THETA (0,.1)

$OMEGA BLOCK(2)
.4    ; OMCL
.04 .4; OMV1
```

```
$ERROR
W=F
IPRED = F
IRES=DV-IPRED
IWRES=0
IF (W.GT.0) IWRES=IRES/W
Y=F*EXP(EPS(1))

$SIGMA
1;SIG

$EST METHOD=1 NOABORT INTERACTION MAXEVAL=5000  PRINT=5 POSTHOC
$COV
$TABLE ID TAD TDOS TIME IPRED IRES IWRES CL V1 CLCR CT01 ONEHEADER NOPRINT
FILE=final.fit
$TABLE ID V1 CL Q2 V2 Q3 V3 CT12 CT01 EX12 EX10 EX25
NOAPPEND NOPRINT FIRSTONLY FILE=param.fit
$TABLE ID TAD TIME DV PRED WRES RES IPRED NPRED NRES
  CPRED CRES CWRES
  EPRED ERES EWRES NPDE
  ESAMPLE=300 SEED=12334 NOAPPEND NOPRINT
  FILE=final.fit
```
