# Supplementary material for: Distribution-based covariate assessment using wasserstein distance in population pharmacokinetic models
Source: Front Pharmacol. 2026 Jul 6;17:1804989. doi: 10.3389/fphar.2026.1804989 (PMC13382220; doi:10.3389/fphar.2026.1804989)
Supplement: Supplementary file 3 [file DataSheet1.pdf]

```
#==========================================================================
=======
# Supplementary R script
# Wasserstein distance based validation from two NONMEM .phi files
# Inputs:
#   - covariates CSV with columns: ID, CLCR, CT01
#   - two NONMEM .phi files (e.g., BASE and FINAL) containing ID and
ETA(1)
# Outputs:
#   - Figure_1_RealData_Covariates_English.png
#   - Figure_2_R2W_English.png
#   - CSV result tables for manuscript integration
#==========================================================================
=======

suppressPackageStartupMessages({
  library(tidyverse)
  library(transport)
  library(patchwork)
  library(scales)
})

#______________________________
# User configuration ----
#______________________________
set.seed(2025)

# Path to covariates file
path_cov <- "mt1_covariates.csv"

# Paths to two .phi files
path_phi_1 <- "base.phi"   # model 1, usually BASE
path_phi_2 <- "final.phi"  # model 2, usually FINAL

# Labels used in plots and tables
label_phi_1 <- "BASE"
label_phi_2 <- "FINAL"

# Permutation settings
n_perm <- 10000

#______________________________
# Helpers ----
#______________________________
read_phi <- function(path) {
  # NONMEM .phi are often space separated and include a first line header
  readr::read_table(path, skip = 1, col_types = cols(.default =
col_guess()))
}

get_eta_cl <- function(phi_df) {
  stopifnot(all(c("ID", "ETA(1)") %in% names(phi_df)))
  phi_df %>%
    transmute(
      ID = as.integer(ID),
      ETA_CL = as.numeric(`ETA(1)`)
    )
}
```

```
fmt_p <- function(p) {
  if (is.na(p)) return("NA")
  if (p < 0.001) return("< 0.001")
  formatC(p, format = "f", digits = 3)
}

wass_perm_2groups <- function(x, y, n_perm = 10000, seed = 1) {
  set.seed(seed)
  x <- as.numeric(x); y <- as.numeric(y)
  x <- x[is.finite(x)]; y <- y[is.finite(y)]
  stopifnot(length(x) >= 2, length(y) >= 2)

  W_obs <- transport::wasserstein1d(x, y, p = 1)

  all <- c(x, y)
  n_x <- length(x)

  W_perm <- replicate(n_perm, {
    s <- sample(all, replace = FALSE)
    transport::wasserstein1d(s[1:n_x], s[(n_x + 1):length(all)], p = 1)
  })

  p_value <- (sum(W_perm >= W_obs) + 1) / (n_perm + 1)

  list(W_obs = W_obs, p_value = p_value, W_perm = W_perm)
}

W_to_zero <- function(x) {
  x <- as.numeric(x)
  x <- x[is.finite(x)]
  transport::wasserstein1d(x, rep(0, length(x)), p = 1)
}

#______________________________
# Load covariates ----
#______________________________
cov <- readr::read_csv(path_cov, show_col_types = FALSE)
names(cov) <- gsub("^X\\.", "", names(cov))

stopifnot(all(c("ID", "CLCR", "CT01") %in% names(cov)))

cov <- cov %>%
  transmute(
    ID   = as.integer(ID),
    CLCR = as.numeric(CLCR),
    CT01 = as.integer(CT01)
  ) %>%
  distinct(ID, .keep_all = TRUE)

cat(sprintf("Patients in covariates file: %d\n", nrow(cov)))

# Define CLCR quartiles once at patient level
cov_q <- cov %>%
  mutate(CLCR_Q = ntile(CLCR, 4)) %>%
  mutate(
    CLCR_Q = factor(
      CLCR_Q,
```

```
      levels = 1:4,
      labels = c("Q1 (low)", "Q2", "Q3", "Q4 (high)")
    )
  ) %>%
  select(ID, CLCR_Q)

#_____________________________
# Load two models ETAs and merge
#_____________________________
eta_1 <- get_eta_cl(read_phi(path_phi_1)) %>% mutate(Model = label_phi_1)
eta_2 <- get_eta_cl(read_phi(path_phi_2)) %>% mutate(Model = label_phi_2)

eta_all <- bind_rows(eta_1, eta_2) %>%
  inner_join(cov, by = "ID") %>%
  left_join(cov_q, by = "ID") %>%
  mutate(
    Model = factor(Model, levels = c(label_phi_1, label_phi_2)),
    CT01_f = factor(CT01, levels = c(0, 1), labels = c("CT01 negative",
"CT01 positive")),
    CLCR_extreme = case_when(
      CLCR_Q %in% c("Q1 (low)", "Q4 (high)") ~ as.character(CLCR_Q),
      TRUE ~ NA_character_
    ),
    CLCR_extreme = factor(CLCR_extreme, levels = c("Q1 (low)", "Q4
(high)"))
  )

#_____________________________
# Tests for Figure 1 style results: CT01 and CLCR extremes ----
#_____________________________
models_for_fig1 <- eta_all

ct01_results <- models_for_fig1 %>%
  group_by(Model) %>%
  group_modify(~{
    df <- .x
    model_name <- as.character(.y$Model[1])

    x0 <- df$ETA_CL[df$CT01 == 0]
    x1 <- df$ETA_CL[df$CT01 == 1]

    tt <- t.test(x1, x0)
    ws <- wass_perm_2groups(
      x1, x0,
      n_perm = n_perm,
      seed = ifelse(model_name == label_phi_2, 101, 100)
    )

    tibble(
      t_stat = as.numeric(tt$statistic),
      p_t    = tt$p.value,
      W      = ws$W_obs,
      p_W    = ws$p_value
    )
  }) %>%
  ungroup()

clcr_results <- models_for_fig1 %>%
```

```
  filter(!is.na(CLCR_extreme)) %>%
  group_by(Model) %>%
  group_modify(~{
    df <- .x
    model_name <- as.character(.y$Model[1])

    x_q1 <- df$ETA_CL[df$CLCR_extreme == "Q1 (low)"]
    x_q4 <- df$ETA_CL[df$CLCR_extreme == "Q4 (high)"]

    tt <- t.test(x_q4, x_q1)
    ws <- wass_perm_2groups(
      x_q4, x_q1,
      n_perm = n_perm,
      seed = ifelse(model_name == label_phi_2, 201, 200)
    )

    tibble(
      t_stat = as.numeric(tt$statistic),
      p_t    = tt$p.value,
      W      = ws$W_obs,
      p_W    = ws$p_value
    )
  }) %>%
  ungroup()

print(ct01_results)

cat("\nCLCR extremes results (Q1 vs Q4):\n")
print(clcr_results)

write.csv(ct01_results, "RealData_CT01_Results_2models.csv", row.names =
FALSE)
write.csv(clcr_results, "RealData_CLCR_Extremes_Results_2models.csv",
row.names = FALSE)

#_____________________________
# Figure 1 in English: ECDFs for CT01 and CLCR extremes, both models ----
#_____________________________
col_ct01 <- c("CT01 negative" = "#2ecc71", "CT01 positive" = "#e74c3c")
col_clcr <- c("Q1 (low)" = "#e74c3c", "Q4 (high)" = "#3498db")

make_ct01_ecdf <- function(model_name) {
  df <- models_for_fig1 %>% filter(Model == model_name)
  ann <- ct01_results %>% filter(Model == model_name) %>% slice(1)

  ggplot(df, aes(x = ETA_CL, color = CT01_f)) +
    stat_ecdf(geom = "step", linewidth = 1.3) +
    scale_color_manual(values = col_ct01) +
    annotate(
      "label",
      x = Inf, y = Inf,
      label = paste0(
        "t = ", sprintf("%.2f", ann$t_stat), "\n",
        "p_t = ", fmt_p(ann$p_t), "\n",
        "W = ", sprintf("%.3f", ann$W), "\n",
        "p_W = ", fmt_p(ann$p_W)
      ),
      hjust = 1.05, vjust = 1.25,
```

```
      fill = "white", size = 3.2
    ) +
    labs(
      title = paste0("Genetic covariate CT01, ", model_name, " model"),
      x = "Individual clearance deviation (ETA CL)",
      y = "Empirical cumulative distribution",
      color = "CT01"
    ) +
    theme_bw(base_size = 11) +
    theme(legend.position = "bottom")
}

make_clcr_ecdf <- function(model_name) {
  df <- models_for_fig1 %>%
    filter(Model == model_name) %>%
    filter(!is.na(CLCR_extreme))
  ann <- clcr_results %>% filter(Model == model_name) %>% slice(1)

  ggplot(df, aes(x = ETA_CL, color = CLCR_extreme)) +
    stat_ecdf(geom = "step", linewidth = 1.3) +
    scale_color_manual(values = col_clcr) +
    annotate(
      "label",
      x = Inf, y = Inf,
      label = paste0(
        "t = ", sprintf("%.2f", ann$t_stat), "\n",
        "p t = ", fmt_p(ann$p_t), "\n",
        "W = ", sprintf("%.3f", ann$W), "\n",
        "p W = ", fmt_p(ann$p_W)
      ),
      hjust = 1.05, vjust = 1.25,
      fill = "white", size = 3.2
    ) +
    labs(
      title = paste0("Physiological covariate CLCR extremes, ",
model_name, " model"),
      x = "Individual clearance deviation (ETA CL)",
      y = "Empirical cumulative distribution",
      color = "CLCR"
    ) +
    theme_bw(base_size = 11) +
    theme(legend.position = "bottom")
}

fig1_ct01_m1 <- make_ct01_ecdf(label_phi_1)
fig1_ct01_m2 <- make_ct01_ecdf(label_phi_2)
fig1_clcr_m1 <- make_clcr_ecdf(label_phi_1)
fig1_clcr_m2 <- make_clcr_ecdf(label_phi_2)

Figure_suppl_1 <- (fig1_ct01_m1 | fig1_ct01_m2) / (fig1_clcr_m1 |
fig1_clcr_m2) +
  plot_annotation(
    title = paste0("Figure 1. Real data: residual covariate signal in ETA
CL for ", label_phi_1, " versus ", label_phi_2, " models"),
    subtitle = "ECDF comparisons illustrate attenuation or removal of
residual covariate related distributional signal",
    theme = theme(
      plot.title = element_text(size = 14, face = "bold", hjust = 0.5),
```

```
      plot.subtitle = element_text(size = 11, hjust = 0.5)
    )
  )

Figure_suppl_1
ggsave("Figure_1_RealData_Covariates_suppl.png", Figure_suppl_1,
       width = 14, height = 10, dpi = 300, bg = "white")

#______________________________
# Figure 2: Wasserstein based R2 across two models (distance to zero) ---
-
#______________________________
r2_w <- eta_all %>%
  group_by(Model) %>%
  summarise(W_to_zero = W_to_zero(ETA_CL), .groups = "drop") %>%
  mutate(
    W_ref = W_to_zero[Model == label_phi_1][1],
    R2_W  = 1 - (W_to_zero / W_ref)
  ) %>%
  select(Model, W_to_zero, R2_W)

cat("\nWasserstein based R2 (reference is first model):\n")
print(r2_w)

write.csv(r2_w, "RealData_R2W_2models.csv", row.names = FALSE)

Figure_suppl_2 <- ggplot(r2_w, aes(x = Model, y = R2_W)) +
  geom_col(fill = "gray60", color = "black", linewidth = 0.3) +
  geom_hline(yintercept = 0, linewidth = 0.4) +
  scale_y_continuous(labels = percent_format(accuracy = 1)) +
  annotate(
    "label",
    x = 1.03, y = max(r2_w$R2_W, na.rm = TRUE),
    hjust = 0, vjust = 1,
    label = paste0(
      "R2W = 1 - Wmodel / Wref\n",
      "Wref is the first model"
    ),
    fill = "white", size = 3.2
  ) +
  labs(
    title = "Figure 2. Wasserstein based R2 across models",
    x = "Model",
    y = "Wasserstein based R2 (relative to first model)"
  ) +
  theme_bw(base_size = 11)

Figure_suppl_2
ggsave("Figure_2_R2W_suppl.png", Figure_suppl_2,
       width = 7, height = 5, dpi = 300, bg = "white")
```
